# IL-17A and Th17 Cells in Lung Inflammation: An Update on the Role of Th17 Cell Differentiation and IL-17R Signaling in Host Defense against Infection

**DOI:** 10.1155/2013/267971

**Published:** 2013-07-17

**Authors:** Hsing-Chuan Tsai, Sharlene Velichko, Li-Yin Hung, Reen Wu

**Affiliations:** Center for Comparative Respiratory Biology and Medicine, University of California, Davis, CA 95616, USA

## Abstract

The significance of Th17 cells and interleukin- (IL-)17A signaling in host defense and disease development has been demonstrated in various infection and autoimmune models. Numerous studies have indicated that Th17 cells and its signature cytokine IL-17A are critical to the airway's immune response against various bacteria and fungal infection. Cytokines such as IL-23, which are involved in Th17 differentiation, play a critical role in controlling *Klebsiella pneumonia* (*K. pneumonia*) infection. IL-17A acts on nonimmune cells in infected tissues to strengthen innate immunity by inducing the expression of antimicrobial proteins, cytokines, and chemokines. Mice deficient in IL-17 receptor (IL-17R) expression are susceptible to infection by various pathogens. In this review, we summarize the recent advances in unraveling the mechanism behind Th17 cell differentiation, IL-17A/IL-17R signaling, and also the importance of IL-17A in pulmonary infection.

## 1. Background and Overview of Th17 Cells and IL-17A

CD4^+^ T cells are central mediators of cellular immunity. For many years, CD4^+^ T cells were classified as either T helper (Th)1 or Th2 cells by their effector cytokines and functions [1, 2]. Th1 cells, which express Interferon-*γ* (IFN-*γ*), are responsible for the control of cellular immune responses and tissue inflammation, whereas Th2 cells, which express IL-4, IL-5, and IL-13, are responsible for the regulation of humoral immunity and allergic disease. The discovery of Th17 cells revolutionized our concept of immunopathology and immune regulation. Th17 cells produce proinflammatory cytokines [3, 4] such as IL-17A, IL-17F, IL-22, IL-26, tumor necrosis factor-*α* (TNF-*α*), chemokine (C–C motif) ligand 20 (CCL20) [5], and granulocyte macrophage colony-stimulating factor (GM-CSF) [6]. Although these cytokines all have proinflammatory features, they act on different target cells and therefore contribute to different diseases [7–9]. Th17 cells have been implicated in a wide variety of inflammatory conditions, such as autoimmune diseases, chronic inflammation, and pathogen infection [10]. 

The differentiation of naive T cells to Th17 cells is regulated by multiple signals. The engagement of TCR receptors (Signal 1) and costimulatory molecules (Signal 2) initiates naive T cell differentiation, and then cytokines produced by the innate immune system (Signal 3) direct the differentiation to particular Th subsets. The proinflammatory cytokines IL-1*β*, IL-6, IL-21, and IL-23, and the anti-inflammatory cytokine, transforming growth factor-*β* (TGF-*β*), coordinate to trigger Th17 cell differentiation in a RAR-related orphan receptor-*γ*t (ROR*γ*t) dependent manner [11, 12].

IL-17A is the signature effector cytokine of Th17 cells and contributes to Th17-mediated diseases. Although first identified in CD4^+^ T cells, IL-17A can also be produced by CD8^+^ T cells [13] and innate cells, such as *γδ*T cells [14, 15], NK1.1-iNKT cells [16], neutrophils [17], and also innate lymphoid cells (ILCs) [18–20]. IL-17A appears to act primarily on nonhematopoietic cells such as endothelial cells [21, 22], epithelial cells [23–25], and fibroblasts [26, 27], due to the restricted expression of one of its receptor subunits, IL-17RC [28, 29]. Systemically, IL-17A and IL-17F have been reported to play a pathogenic role in certain autoimmune diseases, including multiple sclerosis and rheumatoid arthritis [30–32]. However, its role at mucosal surfaces appears to be dualistic. While high expression of IL-17A has been linked to inflammatory diseases of the mucosal surface, such as asthma, cystic fibrosis, and chronic obstructive pulmonary disease (COPD) in the airway, as well as inflammatory bowel disease, it appears to play an important protective role against infection, particularly by extracellular bacterial pathogens [26, 33–40]. Here, we will summarize recent studies on Th17 cell differentiation as well as IL-17R signaling and highlight the role of Th17/IL-17A in pulmonary infection. The roles of innate IL-17A-producing cells in the pulmonary infection will also be discussed. 

## 2. Factors Involved in Th17 Cell Development

Although IL17A production by CD4^+^ T cells was first described 20 years ago, Th17 cells were not recognized as a distinct CD4^+^ cell lineage until 2005 [41, 42]. Th17 cell differentiation has primarily been characterized in the murine system [4]. In mice, IL-6 and TGF-*β* initiate Th17 cell differentiation by activating STAT3 and inducing IL-23R expression. IL-23 is then responsible for Th17 cell maintenance and expansion [11, 43–45]. In addition, Th17 cells also secrete IL-21, an autocrine mechanism to sustain and promote their own differentiation via a STAT3-mediated manner [46, 47]. IL-1*β* was initially thought to play an accessory role in mouse Th17 cell differentiation but recently it has been demonstrated to play a critical role in the early differentiation stages of mouse Th17 cells [48].

However, human Th17 cell differentiation is intrinsically different from murine Th17 because IL-23R is already expressed on human naive T cells, prior to differentiation [49]. IL-1*β* and IL-23 are sufficient to induce human Th17 cells from CD4^+^CD161^+^ cells derived from umbilical cord blood [50], whereas the role of TGF-*β* has been controversial [51, 52]. Recently, it has become clear that TGF-*β* plays an auxiliary role in the suppression of Th1 and Th2 cells [53]. TGF-*β* orchestrates with proinflammatory cytokines to promote Th17 cell differentiation in a dosage-dependent manner [54]. At low concentrations, TGF-*β* induces ROR*γ*t expression and promotes the expression of ROR*γ*t-inducing genes. However, at high concentrations, robust forkhead box P3 (FOXP3) expression induced by TGF-*β* suppresses Th17 cell differentiation by antagonizing ROR*γ*t function [55]. In our laboratory, we also found that human naive T cells responded differentially to the concentration of TGF-*β*, depending on the individual donor (Tsai, HC, unpublished data).

Through cytokine signaling or environmental factors, multiple transcription factors (TFs) are induced to drive Th17 differentiation [56], such as STAT3, Runt-related transcription factor 1 (Runx1) [57], ROR*α*, ROR*γ*t, aryl hydrocarbon receptor (AHR), interferon regulatory factor 4 (IRF4), and basic leucine zipper transcription factor (BATF) [58]. These TFs not only regulate IL-17A expression but the expression of other Th17-associated genes as well. The expression of Th17-signature cytokines, such as IL-17A, IL-17F, and IL-22, is differentially regulated by Th17-lineage transcription factors. ROR*γ*t is the “master regulator” for Th17 differentiation and also directly binds to cis-regulatory elements of the IL17A/F gene [59, 60]. AHR responds to a physiological ligand, tryptophan photoproduct 6-formylindolo[3,2-b]carbazole (FICZ), to promote both IL17A and IL22 expression [61]. The expression of IL17A and IL22 is differentially regulated by TGF-*β*. IRF4 regulates not only IL17A expression [62, 63] but also Th2 cytokine expression [64]. Thus, IRF4 may regulate Th2 and Th17 differentiation by interacting with different transcription factors. The STAT family also plays a vital role in Th17 differentiation. STAT3 is activated by Th17 promoting cytokines (IL-6, IL-21, and IL-23) and directly binds to the promoter of the *Il17a-Il17f* locus, as well as the *Il21* gene [45]. On the other hand, other Stat molecules, including Stat1, activated by IL-27 [65, 66], and Stat5a/b, activated by IL-2 [67], play inhibitory roles in Th17 differentiation. Recently, the reciprocal action of STAT3 and STAT5 on the *Il17a* loci has been reported [68]. STAT3 and STAT5 have been demonstrated to compete for the same binding sites of the *Il17a-Il17f* locus [68]. The relative ratio of STAT3/STAT5 affects the intensity of IL-17A and IL-17F expression in Th17 cell differentiation [68].

The differential regulation of Th17 cytokines also reflects their different roles in physiological conditions and disease pathogenesis [7, 69]. For instance, Yang and colleagues [8] suggested that IL-17A was required to induce EAE, whereas IL-17F was required to induce airway neutrophilia in allergic airway animal models. Additionally, it was demonstrated that IL-22 but not IL-17A was required to protect mice from *Citrobacter rodentium* infection [70].

The understanding of Th17 cell differentiation has been applied to the development of therapies targeted to Th17-mediated autoimmune diseases [71]. Synthetic or natural forms of ROR*γ*t inverse agonists have been studied to suppress IL-17A expression. SR1001, one of the inverse agonists, was shown to be efficacious in the experimental autoimmune encephalomyelitis (EAE) model in rodents [72]. The comprehensive regulation of different Th17-related gene expression urgently needs to be studied for the development of more specific therapy in diseases.

## 3. IL17 Family and Their Receptors

Interleukin-17A was first identified in activated rodent T lymphomas, termed CTLA-8, and subsequently identified in humans in 1995 [73, 74]. At the time, it was noted that IL-17A had a unique structure among the interleukin cytokines. Five related cytokines were subsequently discovered through genome database searches and degenerative RT-PCR techniques [75]. The IL-17A cytokine family members (IL-17A, IL-17B, IL-17C, IL-17D, IL17E/IL-25, and IL-17F) share 20–50% homology at the amino acid level [76]. IL-17F is the most closely related member to IL-17A, and the IL-17F gene is located in the same chromosomal region as *Il17a* in humans (6p12). The resultant protein is approximately 44% homologous to the IL-17A protein and forms as homodimers and heterodimers with IL-17A, and binds a shared receptor heterodimer, IL-17RA/IL-17RC [28, 77–81]. IL-25 is the most distantly related member of the IL-17 family, with only 20% homology to the IL-17A protein. IL-25 also binds to a different receptor heterodimer, IL-17RA/IL-17RB [82]. IL-17B, IL-17C, and IL-17D are less well characterized. IL-17B and IL-17C were reported to be associated with TNF-*α* production and inflammatory arthritis [83]. In recent studies, IL-17C has been demonstrated to bind to IL-17RA/RE and has similar biological functions to IL-17A [84–86]. Similar to IL-17A, Act1 activation is required for these IL-17C-induced responses [84]. In an EAE model, IL-17C deficient mice exhibited less severe disease; this phenomenon demonstrates the pathogenic role of IL-17C in EAE. IL-17C also promoted Th17 responses via IL-17RE signaling in an EAE model [84]. IL-17C was reported to induce the expression of cytokines, chemokines, and antimicrobial peptides by epithelial cells. Overall, IL-17C is important in host defenses against pathogens [85, 86]. IL-17D is preferentially expressed by the nonimmune cells that compose skeletal muscle, adipose and lung tissue. It induces IL-6, IL-8, and GM-CSF expression in endothelial cells and suppresses hematopoiesis [87]. Since Th17 cells express only IL-17A and IL-17F, we will highlight their roles and what is known regarding IL-17R signaling in the following discussion. 

### 3.1. IL-17R Signaling

The IL-17R family is composed of five receptors (IL-17RA-IL-17RE) and the ligand-receptor pairing is not completely understood for all members. Extensive biochemical studies have been executed to characterize IL-17 binding to its receptors [88]. Briefly, IL-17A and IL-17F can form homodimers or heterodimers (IL-17A/A, IL-17A/F, IL-17F/F) to bind to a heteromeric receptor complex composed of IL-17RA and IL-17RC. Surface plasmon resonance (SPR) studies revealed that the different dimers have different affinities for the receptor subunits [28, 77, 89]. X-ray crystallographic, fluorescence resonance energy transfer (FRET) and SPR analyses suggest that IL-17RA homodimers are preassembled as “inactive” receptors on the cell membrane in the absence of ligand binding and that ligand binding shifts the favorability towards the formation of an IL-17RA and IL-17RC heterodimer [78, 79, 89]. 

In 2003, a bioinformatics approach was used to identify a conserved domain present in IL-17RA and the other IL-17 receptor family members, that was distantly related to the TIR domain in Toll-like receptor (TLR) and IL-1 receptor (IL-1R) signaling [90]. Because of the similarity to the TIR domain, this domain, termed the SEFIR (SEF/IL-17R) domain, was proposed to belong to a superfamily with the TIR domain, termed the STIR superfamily. In TLR signaling, the TIR domain mediates the binding of adaptor proteins such as MyD88 and Mal/TRAP to the receptor via homotypic interactions between their respective TIR domains [91, 92]. However, the SEFIR domain lacks the TIR box 3 subdomain and the BB-loop [90], which are critical for the protein-protein interaction of TLR signaling [93]. MyD88 and Mal are not thought to be involved in IL-17 signaling. Although lacking BB loop, a TIR-like loop (TILL) at the C′-terminal side of SEFIR domain in IL17RA, which sequence is homologous to BB loop, may provide the surface for protein-protein interaction [94, 95]. Another SEFIR-domain containing protein, Act1 (also known as CIKS) was later identified as an essential mediator of IL-17 signaling via its interaction with IL-17R [28, 96]. The shRNA knockdown of Act1 expression was shown to attenuate IL-17A signaling in mouse embryonic fibroblasts (MEFs) [97]; likewise, Act1-null mouse embryonic fibroblasts were shown to be unresponsive to IL-17A stimulation [98]. The direct interaction of Act1 and IL-17RA was demonstrated through coimmunoprecipitation experiments and shown to be dependent on the SEFIR domain [97, 98]. Act1 KO mice were shown to have reduced EAE and DSS-colitis induced disease severity, similar to IL-17A KO mice [98]. It was subsequently shown that Act1 can also interact with IL-17RC, as well as IL-17RB, in a SEFIR domain dependent manner [28, 96]. Act1 was also shown to be an essential mediator of IL-25 signaling [96]. Now, it is clear that the CC′ loop of the SEFIR domain is critical for the SEFIR-SEFIR binding [99]. In addition to the SEFIR domain, it has recently been shown that the C-terminal region beyond the SEFIR domain, for both IL-17RA and IL-17RC, is also necessary for the full activity of IL-17A [100]. 

 Act 1 has a TNFR-associated-factor- (TRAF-) binding domain at the amino terminus and a coiled-coil domain containing the SEFIR domain at the carboxyl terminus. TRAF3 and TRAF6 have both been shown to associate with IL-17RA. TRAF6 associates indirectly with IL-17RA through Act1 and, in most cases, positively mediates IL-17A signaling [101]. Recently, TRAF3 has been shown to interact directly with IL-17RA, via a TRAF-binding domain at the distal C-terminus of the receptor's intracellular domain, as well as with the intracellular domain of IL-17RC [102]. Most intriguingly, TRAF3 has been shown to inhibit IL-17A signaling and IL-17 mediated EAE, the first demonstration of a negative regulatory feedback mechanism for IL-17A. Although the mechanism of this negative regulation is not completely clear, it may in part be due to the fact that TRAF3 binding to the distal domain of IL-17RA appears to interfere with Act1-TRAF6 binding to the SEFIR domain of IL-17RA.

In addition, Act1 is also a U-box type E3 ubiquitin ligase and it ubiquitinates TRAF6. The TRAF6 ubiquitination is required for the IL-17A-induced activation of nuclear factor-*κ*B (NF-*κ*B) [103]. The canonical NF-*κ*B pathway is the most well-described downstream signaling pathway of IL-17A. Indeed, IL-17A induces phosphorylation of p65 at Ser^536^; our lab and others have demonstrated p65 and p50 translocation into the nucleus following IL-17A stimulation [23, 104]. Mutation of NF-*κ*B binding sites in the promoter region of the IL-17A target gene, human beta defensin 4 (*DEFB4*), severely attenuates promoter activation in response to IL-17A stimulation in airway epithelial cells [105]. NF-*κ*B is also the major pathway responsible for IL-17 induced early response genes (<4 hours) [104]. However, NF-*κ*B cannot be the sole pathway responsible for IL-17A's effects. For example, in comparison to “classical” NF-*κ*B activating cytokines such as TNF-*α* and IL-1*β*, activation of p65-p50 NF-*κ*B by IL-17A is relatively weak; yet, induction of DEFB4 in airway epithelial cells by IL-17A is much greater than either TNF-*α* or IL-1*β* [23]. It is plausible that it is the synergistic induction of multiple transcription factors including NF-*κ*B, which is responsible for IL-17A's effects. Indeed, other transcription factors, such as AP-1 and C/EBP*δ* (CCAAT/enhancer binding protein *δ*), have been shown to be activated by IL-17A [94, 106]. In addition, all three mitogen activated protein (MAP) kinase pathways, JNK (JUN Ntermainal kinase), ERK (extracellular signal-related kinase) and p38, have been described in the literature as being activated by IL-17A [107]. The relative contribution of the individual pathways appears to depend both on the cell type being studied, as well as on the target gene being studied. In airway epithelial cells specifically, our lab has demonstrated that JAK1/2 and PI3-kinase, Act1/TRAF6/TAK1/NF-*κ*B, and MEK1/2 (MAP kinase kinase1/2)-ERK are all involved in IL-17 mediated gene expression, and that the pathway involved varied depending on the target gene in question [23, 108–110]. Other labs have shown the involvement in p38 in IL-17 mediated IL-6 and IL-8 gene expression by airway epithelial cells as well [111, 112]. Act1 has been shown to be necessary for IL-17A induced NF-*κ*B and C/EBP*δ* activation, as well as JNK and p38 activation [98]. Interestingly, IL-17 induced ERK activation appears to be Act1 independent [98, 104].

 IL-17A utilizes two different methods to increase target gene expression. The first is by transcriptional activation; we have previously demonstrated that this is the case for *DEFB4* and *CCL20* induction by IL-17A in airway epithelial cells using promoter-luciferase reporter assays [105, 109]. The second method of increasing gene expression is by stabilization of the target mRNA via a tristetrapolin/AUUUA-independent mechanism. This has been demonstrated in HeLa cells for both IL-17A induced *CXCL1* and *NFIKBZ *expression [113–115]. The mRNA stabilization pathway appears to be dependent on Act1, but independent of TRAF6, the first demonstration of Act1-dependent, TRAF6-independent IL-17 signaling.

More fine-tuned control of Act1 and IL-17R signaling has been recently described. Act1 exists in multiple phosphorylated forms, which display different functions. In 2010, Act1 was found to be phosphorylated upon IL-17A stimulation [116]. The inducible kinase IKKi (inducible inhibitor of NF-*κ*B (I*κ*B) kinase; also known as IKK*ε*) forms a complex with Act1 and IL-17R and catalyzes the phosphorylation of Act1 at Ser^311^, adjacent to its putative TRAF-binding motif [117]. The phosphorylated form of Act1 appears to have different affinities to various TRAF proteins. Mutation of IKKi or substitution of S311A of Act1 abolished Act1's interaction with TRAF2 and TRAF5, but not TRAF6. This phosphorylated form of Act1 has also been shown to be important for IL17R-Act1-TRAF2/5-mediated mRNA stability. Neither IKKi nor phosphorylation of Ser^311^ on Act1 is required for IL-17A-induced activation of NF-*κ*B. However, IKKi is still responsible for the IL-17A-induced expression of pro-inflammatory genes (*Cxcl1, Cxcl2, Tnf, Il6, *and* Csf3*), resulting in neutrophilia and pulmonary inflammation. A different story is found for other phosphorylated forms of Act1. Three additional serines on human Act1 (Ser^162^ (not phosphorylated by IKKi), Ser^220^, and Ser^233^) and mouse Act1 (Ser^147^, Ser^209^, and Ser^222^) are phosphorylated by IKKi and TBK1 (TANK binding kinase 1, another IKK-related kinases) [118]. TBK1 and IKKi play redundant roles in the phosphorylation of these three sites sites and act to suppress IL-17A-induced activation of NF-*κ*B. In this study, the authors reported that IL-17A-induced Act1 phosphorylation is TRAF6-dependent and serves to suppress IL-17A-induced gene production, such as *ccl20, ccl3, cxcl2*, and *KC*. Interestingly, IKKi appears to regulate IL-17-induced Act1 phosphorylation at different sites via both TRAF6-dependent and TRAF6-independent pathways. More research regarding kinase-mediated Act1 phosphorylation and their specific roles in IL-17A-induced inflammatory response is needed. 

### 3.2. Beyond IL-17RA: IL17-RC and IL-17RD

IL-17RA serves as the common receptor for IL-17 family members, in a manner similar to that of gp130 in IL-6 family signaling. IL-17RA is the most well-characterized IL-17R subunit because of its critical role in IL-17 and IL-25 induced signaling. However, in addition to IL-17RA, IL-17RC and IL-17RD have also been shown to have distinct functions in IL-17-mediated signaling. 

IL-17RC was identified by a homology search of a mammalian expressed sequence tag database and found to share 22% sequence homology with IL-17RA [29, 119]. Unlike IL-17RA, IL-17RC has no obvious TILL structure in its cytoplasmic domain; whether Act1, TRAF6, or other signaling intermediates are recruited to IL-17RC are unclear [88]. Although the CC′ loop, which is responsible for the interaction of Act1/IL-17RA, is also conserved in IL-17RC [99], no direct evidence supports the interaction of Act1 and IL-17RC. Intriguingly, IL-17RA and IL-17RC have strikingly distinct tissue expression patterns. In contrast to IL-17RA, IL-17RC is preferentially expressed in nonimmune cells of the prostate, liver, kidney, thyroid, joints, and lung [77, 119–121]. In term of biological functions, IL-17RA and IL-17RC have differential affinity to IL-17A and IL-17F [28, 77, 89]. In humans, IL-17RA binds to IL-17F with extremely low affinity but IL-17RC has higher affinity binding to IL-17F than IL-17A. In mice, IL-17RA binds both IL-17A and IL-17F but IL-17RC only binds to IL-17F. Therefore, both IL-17RA and IL-17RC are required for IL-17F signaling. With the exception of IL-17RA, IL-17RC has various spliced isoforms and the affinity of IL-17RC splice variants to IL-17A and IL-17F are variable [29, 77]. Since some IL-17RC variants have no affinity to both IL-17A and IL-17F, it is possible that IL-17RC may have other ligands as well. The existence of soluble forms of IL-17RC have been demonstrated in humans but their physiological roles as well as that of the other variants are still unclear. Soluble IL-17RC has been proposed as a decoy receptor to inhibit IL-17R signaling but no evidence supporting this hypothesis yet exists [119]. Although little is known about exactly how IL-17RC participates in signaling, the cytoplasmic tail of the extended SEFIR domain of IL-17RC is essential for functional IL-17A-dependent signaling and IL-17RC knockout mice are susceptible to *Candida albicans *[100]. IL-17RC has also been reported to play a role in a number of human diseases. The levels of IL-17A, IL-17F, IL-17RA, and IL-17RC are also high in the sera and inflamed synovium of patients with rheumatoid arthritis [122–124]. However, the specific role of IL-17RC in these diseases has not been clarified.

IL-17RD was also known as SEF (similar expression to the FGFR) due to its similar expression pattern to the fibroblast growth factor receptor during zebrafish development [90]. The role of IL-17RD in FGF signaling and development is beyond the scope of this review. Previous findings showed that basal IL-17RD expression is higher than IL-17RA; in addition, IL-17A stimulation enhances the expression of other IL-17R family members, but not IL-17RD expression [125]. It has been indicated that IL-17RD may interact with IL-17RA to mediate IL-17A signaling, but the mechanism by which the interaction occurs has not yet been elucidated [126]. Recently, it has been reported that the orphan receptor IL-17RD differentially regulates IL-17A-induced NF-*κ*B and p38 MAPK signaling. IL-17RD utilizes its SEFIR domain to sequester Act1 from interacting with IL-17RA and TRAF6, thereby negatively regulating NF-*κ*B signaling. IL-17RD may therefore act as a basal braking system to prevent IL-17A mediated NF-*κ*B activation. Conversely, IL-17RD promotes IL-17A-induced activation of p38 MAPK to induce the expression of the neutrophil-attractive chemokine, CXCL2, so the net effect of IL-17RD in IL-17A-mediated neutrophilia is unclear [127]. 

## 4. IL-17A-Induced Gene Expression in the Airway

IL-17A acts on a variety of cell types [128]. The best characterized IL-17A-targeted cells are nonimmune cells, such as epithelial cells and mesenchymal cells. In addition, IL-17A also acts on some immune cells. Immune cells express IL-17RA but not IL-17RC, and some studies have described that IL-17 synergized with B cell activation factor to promote B cell survival and proliferation [129] and that IL-17A induced matrix metalloproteinase 9 (MMP-9) expression in monocytes/macrophages [130, 131]. Here, we focus our discussion on the recent discoveries regarding IL-17A-mediated gene expression in airway epithelial cells and other cells in the airway. 

 In the context of airway epithelial cells, IL-17A-targeted genes can be roughly divided in three different categories: antimicrobial molecules, chemokines/cytokines, and adhesion molecules. A number of proteins with antimicrobial properties have been described as being upregulated by IL-17A, including CCL20, DEFB4, MUC5B/AC, S100A7, S100A8, and LCN2/24p3 (lipocalin 2) [25]. These proteins are important for the protective effect of IL-17 against extracellular pathogens. In addition, IL-17A stimulates the production of a number of chemokines and cytokines by airway epithelial cells. Some, such as CXCL1, CXCL2, IL-6, IL-8, KC, and GM-CSF, play a critical role in IL-17A's ability to recruit neutrophils to the airway [118, 125, 132–134]. Others, such as CCL20 and IL-19, have the ability to recruit or influence the differentiation of cells of the adaptive immune system, such as Th17 and Th2 cells, respectively [135, 136]. Additionally, in vitro studies showed that IL-17A induces CCL28 expression in the human airway epithelium, which causes the migration of IgE-secreting B cells [137]. Finally, it has been demonstrated that IL-17A can enhance the proliferation of airway epithelial cells, although the target genes responsible for this effect have yet to be identified [138]. IL-17A can also increase the expression of ICAM-1 in airway epithelial cells and claudin-1 and claudin-2 in intestinal epithelial cells, important adhesion, and cell junction molecules [139, 140]. 

Besides airway epithelial cells, IL-17A also directly acts on airway smooth muscle (ASM) [141] and lung microvascular endothelial cells (LMVECs) [142–144]. IL-17A directly enhances ASM contraction through the IL-17RA/RC complex on the basis of a NF-*κ*B/RhoA/ROCK2 signal cascade in murine models of house dust mite-induced and ovalbumin-induced asthma. IL-17A mediated ASM contraction has also been confirmed in human lung tissue [141]. IL-17RA and IL-17RC are also expressed on the surface of LMVECs and IL-17A significantly induces CXCL1 production in LMVECs. In synergy with IL-1*β* and TNF-*α*, IL-17 also enhances CXCL5 and CXCL8 expression in these cells [144].

 One of the most striking features of IL-17A signaling is its ability to synergize with other proinflammatory cytokines, as well as with TLR signaling pathways. In the literature, it has been reported that TNF-*α*, IL-1*β*, IL-22, Oncostatin M, IFN*γ*, BAFF, and CD40L can all synergize with IL-17A to upregulate IL-17A target genes or their respective target genes [129, 139, 145–147]. In addition, IL-17A also synergizes with TLR2 and TLR4 ligands to increase IL-8 production in a human cystic fibrosis bronchial epithelial cell lines [148]. In an *in vivo* context, this may be where IL-17A induces its most potent effects, within the cytokine milieu of an inflammatory setting to further potentiate the inflammatory response. The mechanism by which this synergism occurs is not yet known, but deserves further study.

## 5. IL-17A in Pulmonary Infection

Numerous studies have identified a protective role of IL-17A in immunity against various infections, including the infection of intracellular [133, 149–151] and extracellular bacteria [152, 153], fungi [154, 155], and even parasites [156]. In murine models of airway infection, IL-17A has been shown to play a critical role in the defense against extracellular bacterial pathogens, such as *K. pneumonia*, and *Pseudomonas aeruginosa *[152, 153, 157]. It has also been reported to play a protective role against intracellular bacterial pathogens, such as *Chlamydia muridarum*, and *Mycoplasma pneumonia*. Although the exact mechanism of this protection is unclear, the deficiency of IL-17 signal or other Th17-associated cytokines in various infection models has shown that neutrophil recruitment is impaired in infected tissue, which is also linked to the reduction of CXC chemokines expression [133, 149–151]. Additionally, Th17 cell response has also been reported to contribute to the mucosal vaccine response against pathogens [158, 159]. Mice vaccinated with antigen from *Mycobacteria tuberculosis* (*Mtb*) provoke a Th17 response, and the CXCL-13 induction by IL-17A is critical in the protection against Mtb infection [159]. IL-17A has also been reported to play a protective role at other mucosal surfaces, with other types of pathogens as well, *Candida albicans* infection in the oral cavity and *Salmonella* dissemination in the intestines [155, 160]. Interestingly, one intracellular respiratory pathogen, *Chlamydia pneumoniae*, has developed a defense against IL-17A signaling. It encodes a protein, CP0236, which binds to the essential IL-17 signaling mediator protein Act1 (also known as CIKS and TRAF3IP2) and sequesters it in bacterial inclusion bodies, leaving it unavailable to mediate IL-17 signaling [161]. The adaption of anti-IL-17 strategies by bacterial pathogens underlines the importance of IL-17A signaling in host defense.

## 6. Innate IL-17A-Producing Cells in Host Defense and Pulmonary Infection

The significance of IL-17A production by innate immune cells in host defense against infection as well as development of autoimmune diseases has been demonstrated and reviewed [162–164]. Cells of the innate immune system are abundant in the skin and at mucosal surfaces and respond rapidly to pathogenic infection, providing the first line of defense. Interestingly, innate IL-17A-producing cells share some characteristics with Th17 cells. For instance,s *γδ*T cells respond to IL-1*β*, IL-18, and IL-23 [165–167] and share some common transcription factors with Th17 cells, such as AHR, ROR*γ*t, and STAT3 [59, 168, 169]. ILCs can be divided into three functionally distinct types; ROR*γ*t is required for the differentiation and maintenance of type 3 ILCs [170, 171]. These ROR*γ*t + ILCs express either IL-17A or IL-22, and some of them express both IL-17A and IL-22 [18–20, 172, 173]. iNKT cells constitutively express IL-23R and ROR*γ*t [174, 175] and produce IL-17A upon stimulation by IL-1*β*, IL-18, IL-23, and TGF-*β* [174, 176, 177]; however, unlike Th17 cells, iNKT cells do not respond to IL-6 stimulation [174]. 

Early IL-17A production by these innate cells provides an initial response to pathogens to recruit neutrophils within 4–8 hours after infection. In the lung, *γδ*T cells have been demonstrated to be the major source of early IL-17A production in response to some infections, such as *K. pneumonia *[178],* M. tuberculosis *[15, 179], and *Mycobacterium bovis* [180]. In the *M. bovis*-infected mouse model, the IL-17A secretion by *γδ*T cells is essential for mature granuloma formation and resolution of infection [180]. IL-17A-producing iNKT cells comprise up to 40% of pulmonary iNKT cells [16] and may also be responsible for the infection with *Streptococcus pneumonia *[181]. In addition, early IL-17A responses may also promote subsequent adaptive immune responses. IL-17A has been reported to induce chemokines to attract Th1 cells into the lung [182, 183], and this secondary response may provide more efficient pathogen clearance. In an EAE model, the depletion of *γδ*T cells is responsible for the development of fewer antigen-specific Th17 cells [165]. Therefore, innate IL-17-producing cells may also enhance or direct the development of later Th17 responses. 

## 7. Summary and Perspectives

To summarize, Th17 effector cytokines such as IL-17 are differentially regulated via multiple transcription factors and play different roles in diseases. Through multiple IL-17R subunits and the posttranslational modification of Act1, IL-17A mediates tissue inflammation and host defense in many facets of signaling regulation. IL-17A induced production of pro-inflammatory cytokines, chemokines, and antimicrobial peptides by multiple cell types in the airway is critical for mounting successful host defense against pathogens. Due to advances in Th17/IL17A research, efforts are now underway to apply some of these findings to the clinical setting, particularly in the setting of autoimmune diseases. A delicate balance is needed to dampen the pathogenic effects of Th17/IL-17A in inflammatory and autoimmune diseases while maintaining the important role it plays in airway host defense. A more comprehensive understanding of Th17 cell differentiation and their functions is urgently needed to provide specific molecular targets to constrain disease-specific cytokine production from Th17 cells but retain other functions of Th17 cells. In addition, the role of innate IL-17A-producing cells in contributing to the resolution of infection and the progression of inflammation cannot be overlooked. Studies on the regulation mechanism of innate-IL-17-producing cells and the clinical relevance of these cells are still limited. A comprehensive understanding of these innate IL-17 cells may be useful in the development of disease therapy. Additionally, a thorough knowledge of cell-type specific IL-17 signaling mechanism also provides alternative therapeutic potentials in IL-17A-mediated diseases. 
